# Antibody-drug conjugates as game changers in bladder cancer: current progress and future directions

**DOI:** 10.3389/fimmu.2025.1591191

**Published:** 2025-05-13

**Authors:** Fei Zhang, Sheng Li

**Affiliations:** ^1^ Department of Urology Surgery, Ningbo No. 2 Hospital, Ningbo, Zhejiang, China; ^2^ Department of Anorectal Surgery, Ningbo No. 2 Hospital, Ningbo, Zhejiang, China

**Keywords:** antibody-drug conjugates (ADC)s, bladder cancer, clinical trial, current landscape, pharma intelligence database

## Abstract

**Introduction:**

Antibody-drug conjugates (ADCs) have emerged as a transformative therapeutic modality in oncology, offering unprecedented precision in targeting tumor cells while sparing healthy tissues. In bladder cancer, a malignancy with high recurrence rates and limited treatment options, ADCs have demonstrated remarkable efficacy by targeting specific tumor-associated antigens such as NECTIN-4 and Human Epidermal Growth Factor Receptor 2 (HER2). This review provides a comprehensive evaluation of the current landscape of ADC-based therapies for bladder cancer, focusing on their mechanisms of action, clinical efficacy, and safety profiles.

**Methods:**

We systematically analyze 232 clinical trials from 2004 to 2025, revealing a significant upward trend in ADC research, particularly following the Food and Drug Administration’s (FDA) accelerated approval of Enfortumab vedotin in 2019.

**Results:**

Our findings highlight the predominance of HER2, NECTIN4, and PD-1 as the most extensively studied molecular targets, with a growing interest in combining ADCs with immune checkpoint inhibitors. Geographically, the United States and China lead in ADC clinical trials, reflecting robust research investment and infrastructure.

**Discussion:**

espite the promising advancements, challenges such as toxicity management, patient stratification, and trial design remain critical. This review underscores the importance of continued innovation in ADC technology and personalized approaches to overcome these limitations, ultimately paving the way for more effective and safer treatment options for bladder cancer patients. The future of ADC therapy in bladder cancer is bright, with immense potential to revolutionize the standard of care and improve patient outcomes globally.

## Introduction

Bladder cancer is the most common cancer of the urinary tract, with more than 600,000 new cases of bladder cancer occurring worldwide each year (1), represents a significant global health burden due to its high incidence and recurrence rates. Clinically, it often manifests with symptoms such as hematuria, dysuria, and urinary frequency (2), which, while indicative, frequently lead to delayed diagnosis and suboptimal patient outcomes. The prognosis of bladder cancer is critically dependent on early detection and precise therapeutic intervention (3), traditional treatments mainly rely on surgery, chemotherapy, and radiation, but their effectiveness is limited, especially for advanced-stage patients who have poor prognosis. In recent years, with the development of precision medicine, immune checkpoint inhibitors have improved the prognosis of some patients. However, their response rate is only 20%-30%, and resistance is easily developed (4). Therefore, there is an urgent need to develop new treatment strategies.

In recent years, ADCs have emerged as a transformative therapeutic modality in oncology, offering a promising avenue for the treatment of bladder cancer. ADCs combine the specificity of monoclonal antibodies with the potent cytotoxicity of chemotherapeutic agents, enabling targeted delivery of payloads to tumor cells while sparing healthy tissues. This unique mechanism of action has led to significant advancements in the treatment landscape, particularly for cancers that are resistant to conventional therapies (5). In bladder cancer, ADCs have demonstrated remarkable efficacy by targeting specific tumor-associated antigens, such as nectin-4 and HER2, thereby inducing tumor cell apoptosis and inhibiting metastasis. For example, Enfortumab vedotin (targeting NECTIN-4) achieved a median overall survival of 12.9 months in the EV-301 trial (vs. 9.0 months with chemotherapy) (6). Similarly, trastuzumab deruxtecan, a HER2-directed ADC, has demonstrated promising antitumor activity in HER2-expressing bladder cancer (7), offering a potential therapeutic option for this subset of patients.

Despite these promising developments, several challenges persist in the clinical application of ADCs for bladder cancer. Key issues include the durability of therapeutic responses, the management of off-target toxicities, and the heterogeneity of antigen expression across different patient subtypes (8). Moreover, the optimal integration of ADCs with existing treatment paradigms, such as immune checkpoint inhibitors and chemotherapy, remains an area of active investigation (9). Addressing these challenges is crucial for maximizing the therapeutic potential of ADCs and improving patient outcomes.

This study provides a comprehensive evaluation of the current landscape of ADC-based therapies for bladder cancer, with a focus on their mechanisms of action, clinical efficacy, and safety profiles. We systematically review ongoing clinical trials, highlighting the most promising ADC candidates and their respective targets. Furthermore, we explore emerging strategies to enhance the precision and durability of ADC therapies, including the development of novel linkers, payloads, and antibody engineering techniques. By synthesizing the latest clinical data, this review aims to offer valuable insights into the future directions of ADC research and their potential to revolutionize bladder cancer treatment. Our findings underscore the importance of continued innovation in ADC technology and the need for personalized approaches to overcome the limitations of current therapies, ultimately paving the way for more effective and safer treatment options for patients with bladder cancer.

## Method

**Figure 1:**
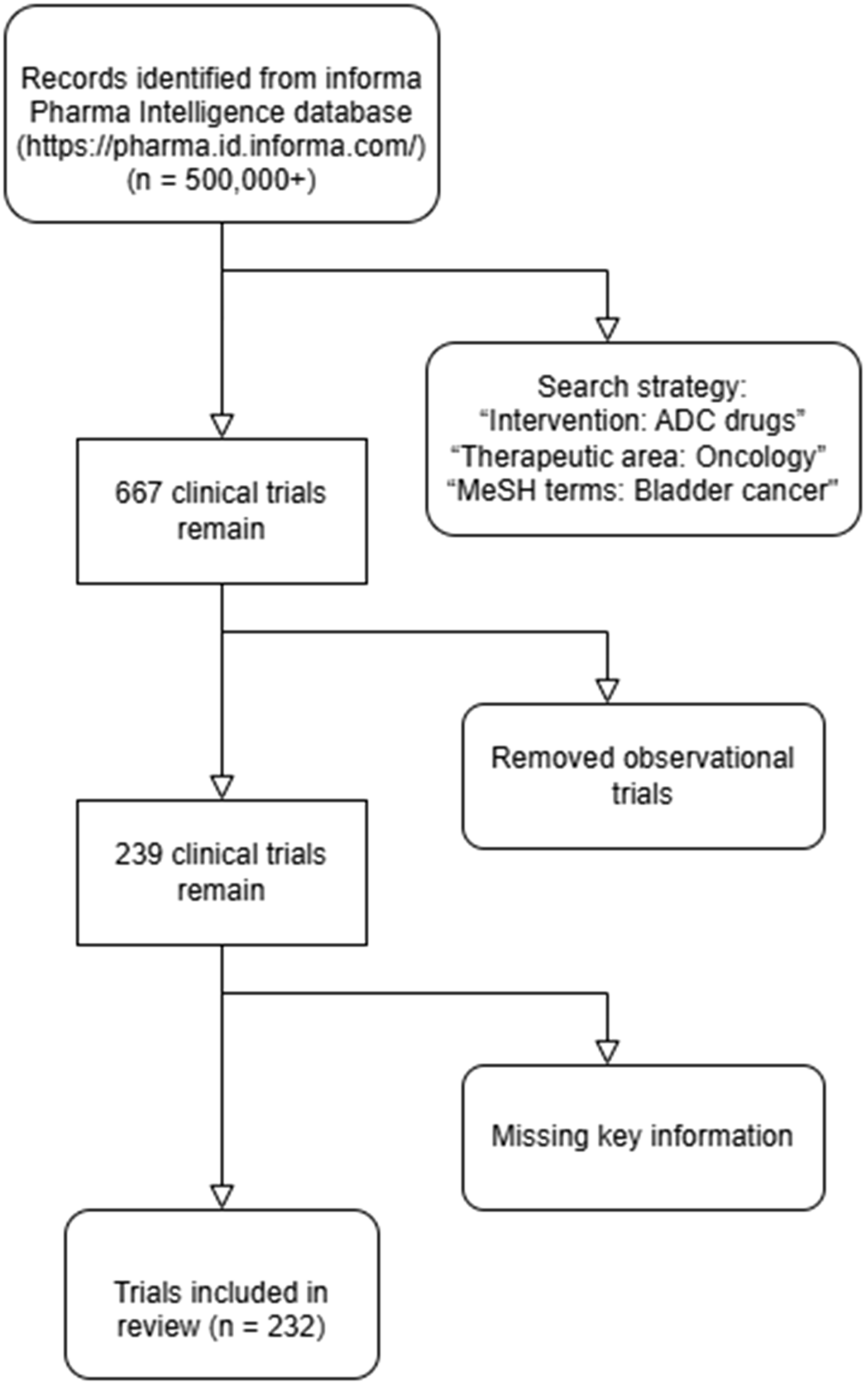
Flowchart of clinical trial selection for ADC therapies in bladder cancer.

To comprehensively evaluate the current landscape of ADC therapies in bladder cancer, we conducted a systematic search of the Informa Pharma Intelligence database (https://pharma.id.informa.com/) (10), a globally recognized repository for clinical trial data and drug development information. The search was performed on January 20, 2025, using a combination of carefully selected keywords and Medical Subject Headings (MeSH) terms to ensure a robust and inclusive retrieval of relevant studies. Specifically, the search strategy included the following terms: “Intervention: ADC drugs,” “Therapeutic area: Oncology,” and “MeSH terms: Bladder cancer.” This approach was designed to capture all publicly available clinical trials investigating ADC therapies in bladder cancer, encompassing a wide range of targets, mechanisms, and developmental stages. (**Figure 1**).

## Results

Our initial search yielded 232 clinical trials meeting the inclusion criteria. (**Supplementary Table S1**). Each trial was meticulously analyzed to extract key data points, including trial identification number (ID), drug name, molecular target, mechanism of action, trial phase (Phase I, II, III or IV), outcome status (completed, ongoing, or terminated), and the country or region where the trial was conducted. This granular level of data collection allowed us to construct a comprehensive overview of the global ADC research landscape in bladder cancer.

**Figure 2:**
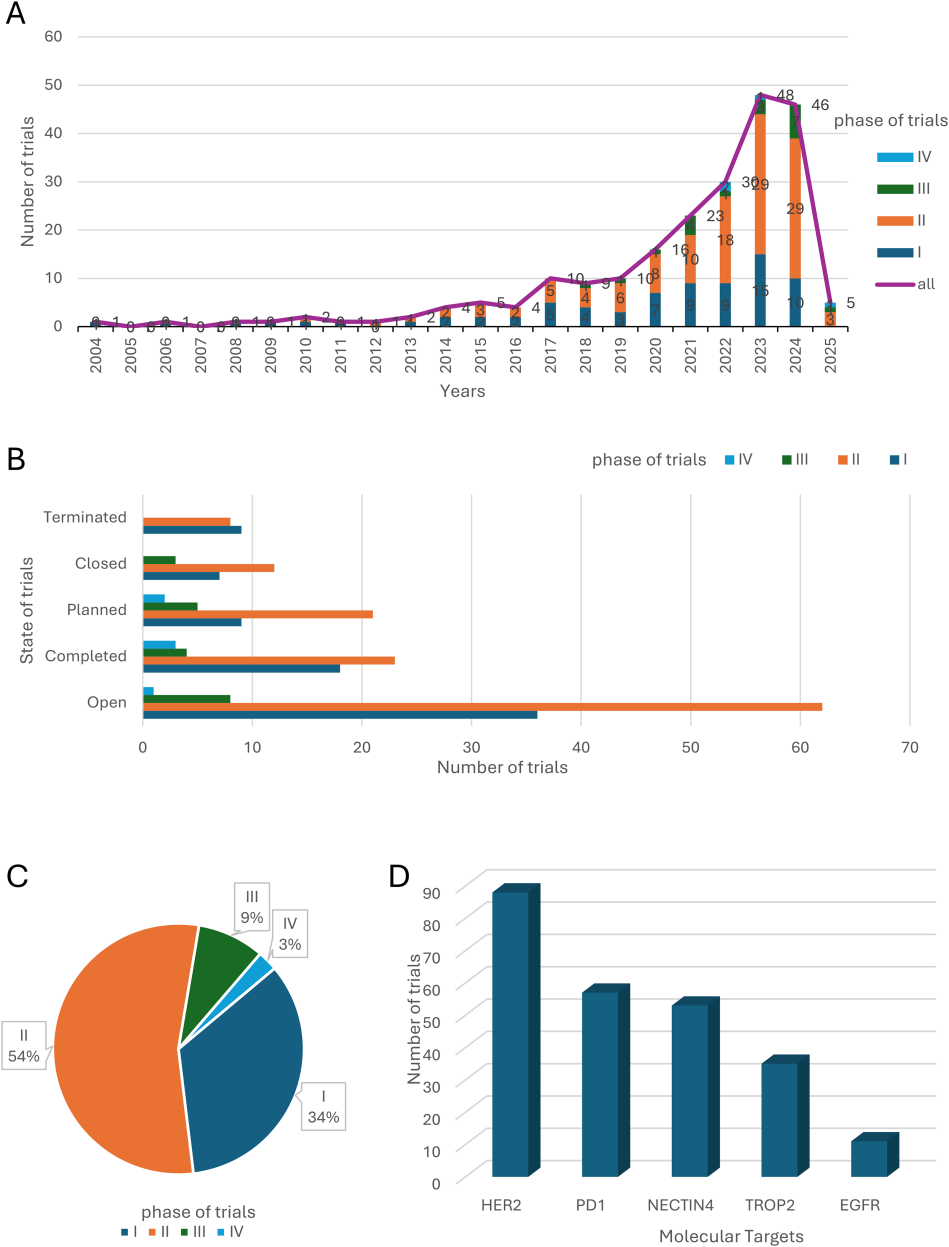
Clinical Trial Landscape of ADC Therapies for Bladder Cancer. **(A)** The number and phase of clinical trials worldwide from 2004 to 2025. **(B)** The clinical states of clinical trials worldwide. **(C)** Clinical phases of clinical trials worldwide. **(D)** The 5 most common drug targets and the corresponding number of clinical trials.

From 2004 to 2025, the landscape of clinical trials investigating ADCs in bladder cancer has undergone a remarkable evolution, characterized by a steady and significant upward trend in trial numbers. This growth trajectory underscores the increasing recognition of ADCs as a transformative therapeutic strategy in oncology. Among 232 trials, several pivotal studies have defined the clinical utility of ADCs: The Phase III EV-301 trial (NCT03474107) (6) established enfortumab vedotin (anti-NECTIN-4) as a standard-of-care for refractory metastatic urothelial carcinoma (mUC), demonstrating a median overall survival (mOS) of 12.9 months versus 9.0 months with chemotherapy (HR=0.70; p<0.001). Sacituzumab govitecan (anti-TROP2) in the TROPHY-U-01 trial (NCT03547973) achieved an objective response rate (ORR) of 27% in heavily pretreated mUC patients (11), leading to accelerated FDA approval. The Phase Ib/II EV-103 trial (NCT03288545) showed that combining enfortumab vedotin with pembrolizumab (anti-PD-1) achieved a 64.5% ORR in cisplatin-ineligible patients, highlighting synergistic potential (12). These trials collectively underscore the transformative efficacy of ADCs, though safety challenges persist (**Table 1**). Notably, the pace of clinical trial initiation accelerated dramatically after 2020, with an estimated 60 trials projected to be active by 2025 (**Figure 2A**). This surge reflects both the growing scientific interest in ADC-based therapies and their entry into a phase of rapid development, driven by advancements in molecular targeting and drug delivery technologies. The pivotal moment in this field came in 2019, when FDA granted accelerated approval to Enfortumab vedotin, the first ADC drug specifically indicated for bladder cancer. This landmark approval not only validated the therapeutic potential of ADCs but also catalyzed a wave of research and development efforts aimed at expanding their application in bladder cancer treatment.

**Table 1:** Key clinical studies in the development of ADC drugs for bladder cancer.

ADC Drug	Target	Trial (Phase)	Patient Population	Efficacy	Key Safety Concerns
Enfortumab vedotin	NECTIN4	EV-301 (III)	Refractory mUC	mOS 12.9 mo (vs. 9.0 mo chemo)	Rash (50%), Peripheral neuropathy (40%)
Sacituzumab govitecan	Trop-2	TROPHY-U-01 (II)	Platinum/PD-1-refractory mUC	ORR 27%, mPFS 5.4 mo	Neutropenia (34%), Diarrhea (10%)
Enfortumab + Pembrolizumab	NECTIN4/PD-1	EV-103 (Ib/II)	Cisplatin-ineligible mUC	ORR 64.5%	Fatigue (32%), Pruritus (25%)

Analysis of trial status further supports the optimism surrounding ADC therapies. The majority of trials are either “ongoing” or “completed,” with very few being terminated or closed prematurely (**Figure 2B**). This low attrition rate suggests a high degree of feasibility and therapeutic potential for ADC drugs in bladder cancer. Data from completed trials have provided preliminary evidence on the safety and efficacy of these agents, while the large number of active trials reflects sustained investment and a strong belief in the future of ADC-based treatments. Despite the inherent complexities of ADC design and development, their unique ability to deliver cytotoxic payloads directly to tumor cells while sparing normal tissues positions them as a highly promising approach for bladder cancer therapy.

A detailed analysis of trial phases reveals that the majority of ADC-related clinical trials are concentrated in Phase I (34%) and Phase II (54%), with fewer trials advancing to Phase III (9%) or Phase IV (3%) (**Figure 2C**). This distribution highlights the relatively early stage of ADC development in bladder cancer, with only a limited number of candidates progressing to large-scale validation and regulatory approval. The challenges associated with ADC development, including their structural complexity, toxicity management, and difficulties in patient recruitment, likely contribute to this pattern. Nevertheless, the high proportion of trials in early phases also indicates a vibrant and dynamic research environment, with numerous investigational agents being actively explored.

The distribution of the top 5 molecular targets in the global ADC clinical trials in bladder cancer (**Figure 2D**) showed that HER2, NECTIN4, immune checkpoints (PD-1), TROP2 and epidermal growth factor receptor (EGFR) were the most widely studied targets. These targets were selected based on their tumor-selective expression and internalization ability. Notably, PD-1 and EGFR are not ADC targets per se but are frequently combined with ADCs in therapeutic regimens. HER2 is a transmembrane receptor tyrosine kinase widely expressed in a wide range of solid tumors, and its high expression in bladder cancer correlates with tumor aggressiveness and poor prognosis. HER2-based ADC drugs such as Disitamab vedotin (RC48) (13) have entered clinical trials and demonstrated good efficacy and safety in some bladder cancer patients. NECTIN4 is a cell adhesion molecule that is mainly expressed in embryonic tissues, with low expression in adult normal tissues, but shows high expression in bladder cancer, making it an ideal ADC target. Enfortumab vedotin is a representative ADC targeting NECTIN4 and is currently approved by the FDA for use in patients with locally advanced or metastatic uroepithelial carcinoma after prior platinum-based chemotherapy and immunotherapy (12). TROP2 is a transmembrane glycoprotein that is highly expressed in a variety of solid tumors and has an important role in tumor cell signaling, proliferation and migration. Sacituzumab govitecan, an ADC targeting TROP2, has been used in the treatment of triple-negative breast cancer and is now also under clinical evaluation in bladder cancer (11). PD-1 is an immune checkpoint molecule expressed on the surface of T-cells, which plays an important role in the suppression of autoimmunity and the maintenance of immune homeostasis. Although PD-1 itself is not an ADC target, blockade of its ligand, PD-L1, in combination with ADC has shown synergistic antitumor effects in several studies (14). EGFR is a classical receptor tyrosine kinase that is widely involved in the processes of cell proliferation, differentiation, and survival. In bladder cancer, high expression of EGFR is closely associated with tumor progression. Although EGFR itself is less frequently used as a direct target of ADCs, as an important node of the RTK pathway, its small molecule inhibitors are often used in combination with ADCs to enhance therapeutic efficacy or overcome drug resistance (15).

In addition to the above established targets, with the deepening of the understanding of tumor heterogeneity and drug resistance mechanisms, researchers have been exploring novel ADC targets with the aim of improving therapeutic precision and broad-spectrum. Emerging targets, such as B7-H3 (CD276) and ERBB3 (HER3), have received extensive attention due to their specific expression and cancer-promoting mechanisms in bladder cancer. B7-H3, a member of the B7 immunoregulatory family, is a transmembrane glycoprotein that is widely used as an immunoregulator in bladder cancer. It is a transmembrane glycoprotein widely present on the cell surface of many solid tumors. B7-H3 is a transmembrane glycoprotein widely present on the cell surface of many solid tumors, and its expression rate in uroepithelial carcinoma is as high as 60-80%, and is associated with poor prognosis, making it a dual-acting target with the potential of “direct tumor killing” and “immunosuppression relief”. DS-7300a (16), a representative drug, is currently undergoing multi-center, dose-escalation phase I clinical trials. ERBB3 is a member of the EGFR family, and although its tyrosine kinase activity is weak, it can participate in the activation of multiple oncogenic signaling pathways through the formation of a heterodimer with HER2, and especially plays a central role in the PI3K/AKT pathway. bladder cancer is expressed at a high frequency, and its activation is closely related to tumor proliferation, invasion, and treatment resistance. The results of the relevant phase II clinical study of its representative drug T-Dxd have been published in the European Society of Medical Oncology (ESMO) 2024, demonstrating strong clinical activity and long-lasting clinical benefits (7).

**Figure 3:**
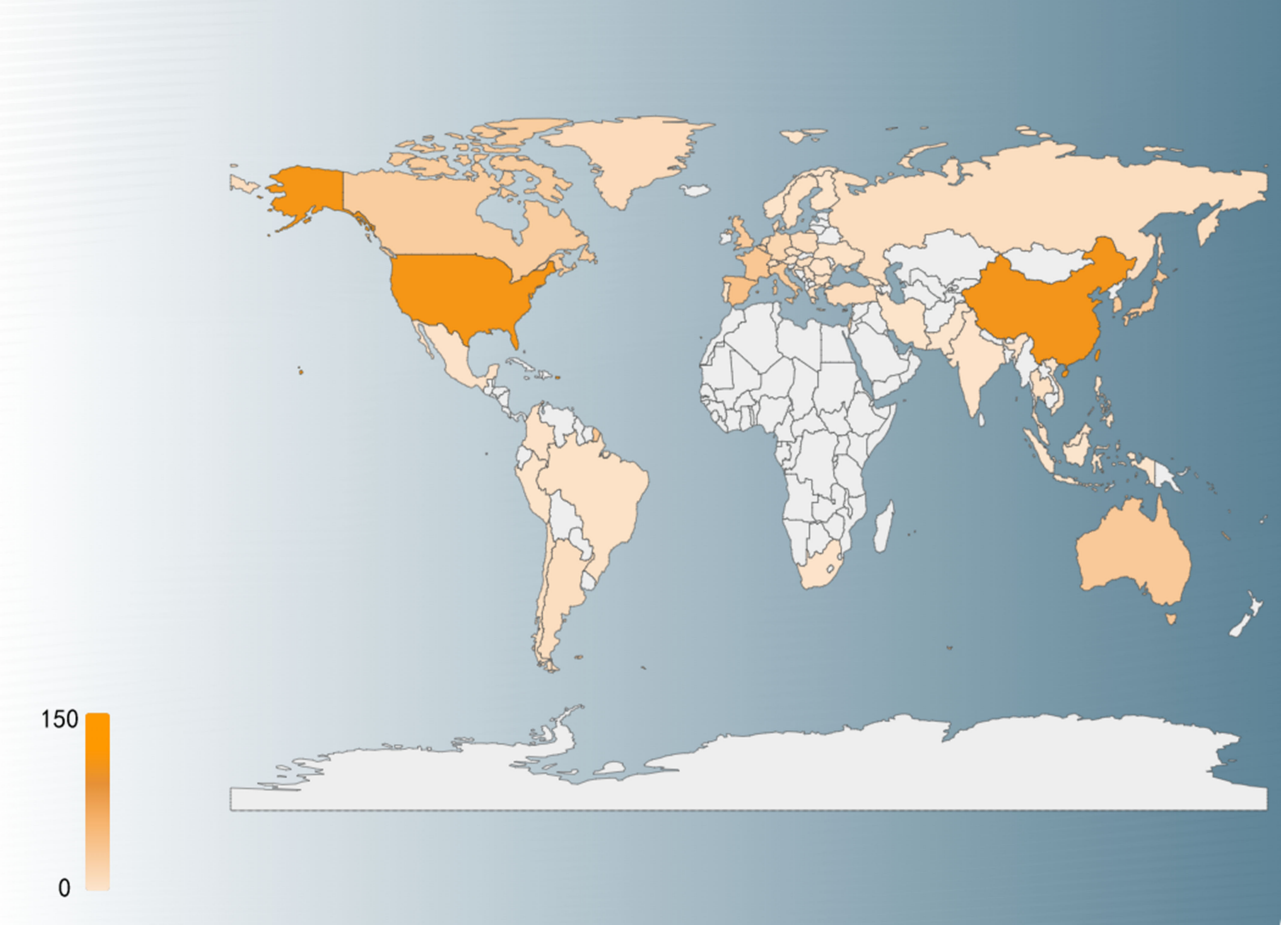
Country distribution of global clinical trials, with darker red representing a higher number of clinical trials.

Geographically, the distribution of ADC clinical trials highlights the leading roles of the United States and China, both of which have surpassed 100 trials each (**Figure 3**). These regions are followed by European countries (e.g., Spain, France), Australia, and East Asian nations (e.g., Japan, South Korea). This geographical pattern reflects the interplay between healthcare infrastructure, research investment, and regulatory frameworks in shaping the development of ADC therapies. As global research collaboration intensifies and emerging markets enhance their capabilities, the application of ADC drugs in bladder cancer treatment is poised to expand further, offering hope for improved outcomes for patients worldwide.

## Discussion

In recent years, ADCs have emerged as a groundbreaking therapeutic modality in the treatment of bladder cancer, particularly for metastatic or refractory urothelial carcinoma (UC), where treatment options have historically been limited. The remarkable progress in this field is exemplified by the development of Enfortumab Vedotin (EV), an ADC targeting Nectin-4, which has redefined the therapeutic landscape for advanced bladder cancer. The pivotal Phase III EV-301 trial (2021) demonstrated that EV significantly improved overall survival compared to traditional chemotherapy (12.9 vs. 9.0 months) (17), leading to its FDA approval as the first ADC for bladder cancer. This landmark achievement not only validated the efficacy of ADCs but also paved the way for their expanded use, including first-line combination therapy with immune checkpoint inhibitors in the ongoing EV-302 trial. The success of EV underscores the transformative potential of ADCs in addressing unmet clinical needs and improving outcomes for patients with advanced disease.

Another notable advancement is Sacituzumab Govitecan (SG), an ADC targeting Trop-2, which has shown promising efficacy in heavily pretreated patients. In the TROPHY-U-01 trial (2021), SG achieved an objective response rate (ORR) of 27% in patients who had failed both platinum-based chemotherapy and immunotherapy (11), leading to its accelerated FDA approval for third-line treatment. This achievement highlights the potential of ADCs to provide meaningful clinical benefits even in later-line settings, where therapeutic options are scarce.

The integration of ADCs with immune checkpoint inhibitors represents a new frontier in bladder cancer treatment. Early clinical data from the EV-103 trial, which combined EV with PD-1 inhibitors, reported an ORR of 64.5%, suggesting synergistic effects that could further enhance therapeutic outcomes (14). This combination strategy is rapidly gaining traction as a promising approach to overcome resistance and improve response rates. Additionally, the exploration of novel ADCs, such as HER3-DXd, is expanding the scope of targetable pathways and offering new hope for patients with diverse molecular profiles (18). These advancements collectively illustrate the immense clinical potential of ADCs in bladder cancer, providing a robust foundation for personalized treatment strategies and informing future drug development.

ADCs have shown remarkable efficacy in the treatment of malignant tumors such as bladder cancer, but their toxicity is still one of the major challenges limiting their clinical application (19). The toxicity associated with ADCs is mainly attributed to a number of factors, such as widespread expression of targeted antigens, lack of linker stability, high cytotoxic efficiency, and individual metabolism differences. First, many tumor antigens are also expressed in normal tissues to varying degrees, leading to the possibility of off-target toxicity in normal tissues while attacking tumor cells (20), e.g., the expression of NECTIN-4 in skin tissues explains ADC-associated rashes and itchy side effects. Second, the stability of the linker is critical to the safety of ADCs; if the linker breaks prematurely in the circulation, free cytotoxicity may damage non-tumor cells and increase the risk of systemic toxicity, Sacituzumab Govitecan, a TTROP-2-targeted ADC with an SN-38 payload, is associated with a high incidence of neutropenia and diarrhea associated with a high incidence of neutropenia and diarrhea (21), reminiscent of its parent drug irinotecan. In addition, the cytotoxins carried by ADCs (e.g., MMAE, DXd, etc.) are themselves extremely cytotoxic, and even small exposures to non-tumor tissues can lead to serious adverse effects such as peripheral neuropathy, bone marrow suppression, and hepatotoxicity (22). Differences in liver and kidney function, metabolic enzyme levels, and immune status of individual patients may also affect the clearance efficiency of ADCs *in vivo*, further exacerbating the individual differences in toxicity.

Despite these successes, several challenges must be addressed to fully realize the potential of ADCs in bladder cancer treatment. Toxicity management remains a critical concern (20), as the potent cytotoxic payloads of ADCs can lead to adverse effects that limit their therapeutic window. Optimizing drug design, including the development of more stable linkers and less toxic payloads, will be essential to improve safety profiles. Additionally, patient stratification based on biomarker expression and tumor biology is crucial to ensure that ADCs are administered to those most likely to benefit. The design of clinical trials also requires careful consideration, particularly in terms of patient recruitment, endpoint selection, and the incorporation of real-world evidence to validate findings from controlled studies.

Looking ahead, the future of ADC therapy in bladder cancer is bright, with immense potential to fill critical gaps in the treatment of advanced disease. Through continued innovation in drug design, exploration of combination therapies, and strengthened international collaboration, ADCs are poised to become a cornerstone of bladder cancer treatment. These efforts will not only enhance therapeutic efficacy but also provide patients with improved survival benefits and quality of life. However, overcoming the existing challenges will require a multidisciplinary approach, integrating insights from basic science, clinical research, and regulatory science. By addressing these hurdles, the field can unlock the full potential of ADCs, transforming the outlook for patients with bladder cancer and setting a new standard of care in oncology.

## Data Availability

The original contributions presented in the study are included in the article/**Supplementary Material**. Further inquiries can be directed to the corresponding author.
